# A temporal analysis of perioperative complications following COVID-19 infection in patients undergoing lumbar spinal fusion: When is it safe to proceed?

**DOI:** 10.1016/j.xnsj.2023.100262

**Published:** 2023-08-11

**Authors:** Justin P. Chan, Henry Hoang, Sohaib Z. Hashmi, Yu-Po Lee, Nitin N. Bhatia, On behalf of the N3C Consortium

**Affiliations:** Department of Orthopaedic Surgery, University of California Irvine, 101 The City Dr S, Orange, CA 92868, United States

**Keywords:** COVID-19, Coronavirus, SARS-CoV2, Lumbar fusion

## Abstract

**Background Context:**

COVID-19 has been shown to adversely affect multiple organ systems, yet little is known about its effect on perioperative complications after spine surgery or the optimal timing of surgery after an infection. We used the NIH National COVID Cohort Collaborative (N3C) database to characterize the risk profile in patients undergoing spine surgery during multiple time windows following COVID-19 infection.

**Methods:**

We queried the National COVID Cohort Collaborative, a database of 17.4 million persons with 6.9 million COVID-19 cases, for patients undergoing lumbar spinal fusion surgery. Patients were stratified into those with an initial documented COVID-19 infection within 3 time periods: 0 to 2 weeks, 2 to 6 weeks, or 6 to 12 weeks before surgery.

**Results:**

A total of 60,541 patients who underwent lumbar spinal fusion procedures were included. Patients who underwent surgery within 2 weeks of their COVID-19 diagnosis had a significantly increased risk for venous thromboembolic events (OR 2.29, 95% CI 1.58–3.32), sepsis (OR 1.56, 95% CI 1.03–2.36), 30-day mortality (OR 5.55, 95% CI 3.53–8.71), and 1-year mortality (OR 2.70, 95% CI 1.91–3.82) compared with patients who were COVID negative during the same period. There was no significant difference in the rates of acute kidney injury or surgical site infection. Patients undergoing surgery between 2 and 6 weeks or between 6 and 12 weeks from the date of COVID-19 infection did not show significantly elevated rates of any complication analyzed.

**Conclusions:**

Patients undergoing lumbar spinal fusion within 2 weeks from initial COVID-19 diagnosis are at increased risk for perioperative venous thromboembolic events and sepsis. This effect does not persist beyond 2 weeks, however, so it may be warranted to postpone non-urgent spine surgeries for at least 2 weeks following a COVID-19 infection or to consider a more aggressive VTE chemoprophylaxis regimen for urgent surgery in COVID-19 patients.

## Introduction

There remains a lack of consensus on the safe timing of elective spine surgery following a COVID-19 infection. COVID-19 has been shown to adversely affect multiple organ systems, yet relatively little is known about its effect on perioperative complications after spine surgery or the optimal timing of elective surgeries after an infection. The impact of elective surgery cancellations on spine surgery has been profound. A survey of AO spine members in 2020 found that only 18.5% of respondents were still performing elective surgery, with most surgeons canceling between 76% and 100% of their cases each week [1]. A subsequent 2021 study found that the percentage of respondents performing elective surgery had increased to 67.6% but that the majority were still having to cancel up to 25% of cases each week due to COVID-19-related delays [2]. The consequences of delaying spine surgery are also not inconsequential. Conditions such as cervical myelopathy can irreversibly progress with stepwise declines, lumbar motor, or sensory deficits can become permanent if left untreated for long periods, and even radicular pain can cost significant time off work and lost productivity [3]. Delays in single-level lumbar fusion surgery specifically have previously been demonstrated to result in a 10-fold increase in mortality, longer operative times, higher rates of intraoperative bleeding, deep venous thrombosis, and pulmonary embolism, return to the operating room, sepsis, stroke, renal insufficiency, urinary tract infection, pneumonia, and surgical site infections [4].

Early in the pandemic, the rationale for delaying elective surgery was due in large part to the risk of transmitting COVID-19 to others [5]. Other patients in the hospital were vulnerable through direct exposure, as well as through contamination of hospital and operating room surfaces and equipment. Staff members were also placed at increased risk, particularly anesthesiologists performing intubation due to the risk of aerosolizing small virus-containing particles [5]. Surgeons were also exposed for long periods of time during the procedure. As vaccinations became widely available and more effective at preventing serious complications and hospitalization, the risk to others has partially abated. As a result, the decision of whether to postpone surgery and for how much time is increasingly falling to surgeons. This presents significant moral and ethical implications for spine surgeons trying to determine the priority and urgency of surgery [6].

Multiple anesthesiology societies have issued joint statements on the timing of elective surgery after COVID-19 infection recommending delay of elective surgery for seven weeks after infection, and longer for patients with ongoing symptoms [7,8]. These recommendations are not specific to orthopedic surgery or to spine surgery, however, none of the professional orthopedic surgery or spine societies have provided recommended practice guidelines with regards to this question either, due to a lack of evidence in this area. In this study, we, therefore, set out to characterize the perioperative complications after spine surgery using a nationally representative sample of patients undergoing spine surgery at varying time points after documented COVID-19 infection.

## Methods

We queried the National COVID Cohort Collaborative (N3C) for patients undergoing lumbar spinal fusion. This database, maintained by the National Institutes of Health (NIH) National Center for Advancing Translational Sciences (NCATS), contains deidentified patient data on 17.4 million persons with 6.9 million COVID-19 cases from over 60 healthcare institutions across the United States [9].

Patients were stratified into those with an initial documented COVID-19 infection within 3 time periods: 0 to 2 weeks, 2 to 6 weeks, or 6 to 12 weeks before surgery. All data collection was performed in the N3C Data Enclave Palantir platform. We analyzed data retrospectively from September 2020 to March 2023. Patients with a positive COVID-19 test were identified using the *International Statistical Classification of Diseases and Related Health Problems, Tenth Revision* (ICD-10) code ICD-10-U07.1. We then screened patients who underwent a lumbar spinal fusion procedure as identified by current procedural terminology (CPT) codes. A full list of the codes can be found in Supplementary Table 1. The search was then narrowed to identify patients who underwent surgery within 12 weeks from the date of initial COVID-19 diagnosis. We then searched for adverse events which occurred within 90 days of surgery. The adverse events analyzed were venous thromboembolic events (VTE), sepsis, surgical site infection, 30-day mortality, and 1-year mortality. A list of ICD-10 codes used to identify each complication can be found in Supplementary Table 2.

The risk of each complication was reported as an odds ratio with a 95% confidence interval using patients who underwent lumbar spinal fusion but did not have a COVID-19 diagnosis within the 6 weeks before surgery as the control group. Descriptive statistics were also performed for demographic information and perioperative complications. Categorical variables were compared with chi-square tests and continuous variables were compared with independent samples *t* tests. Statistical significance was defined as p<.05.

## Results

A total of 60,541 patients who underwent lumbar spinal fusion procedures were included. Baseline characteristics are shown in Table 1, however demographic data including smoking status and comorbidities, were not available for all patients due to incomplete data merging from each contributing healthcare institution. Consequently, the total number in each category varies slightly. The mean age (SD) was 62.56 (14.52) for the COVID-19 positive group and 62.21 (15.10) for the COVID-19 negative group and was not significantly different between groups. Age was comparable between the 2 study groups with a higher proportion of women in the COVID-19 positive group (52.43% vs. 50.24%, p=.002). Body mass index was also comparable between groups although slightly higher on average in the COVID-19 positive group (30.97±7.02 vs. 30.30±7.06, p<.001). When assessing comorbidities, there was a higher proportion of diabetes (35.48% vs. 26.23%, p<.001) and hypertension (75.20% vs. 64.83%, p<.001) in the COVID-19 positive group.

**Table 1:** Comparison of patient demographics and medical comorbidities between COVID-19 Positive and COVID-19 Negative cohorts

Characteristic	COVID-19 positive	COVID-19 negative	p-value
Age (years) (mean ± SD)	62.56±14.52	62.21±15.10	.089
Gender, n (%)			.002
Female	3,186 (52.43)	22,309 (50.24)	
Male	2,891 (47.57)	22,093 (49.76)	
Ethnicity, n (%)			<.001
Caucasian	4,698 (77.31)	35,327 (79.56)	
Black or African American	934 (15.37)	6,102 (13.74)	
Asian	53 (0.87)	393 (0.89)	
Other	392 (6.45)	2,580 (5.81)	
BMI (mean ± SD)	30.97±7.02	30.30±7.06	<.001
Smoking status			.064
Nonsmoker	2,693 (73.12)	19,707 (74.46)	
Current or former smoker	990 (26.88)	6,761 (25.54)	
Data unavailable	2,394	17,934	
Comorbidities			
Diabetic	2,156 (35.48)	11,647 (26.23)	<.001
Nondiabetic	3,921 (64.52)	32,757 (73.77)	
Hypertension	4,570 (75.20)	28,788 (64.83)	<.001
No hypertension	1,507 (24.80)	15,616 (35.17)	

Values are reported as n (%) unless otherwise specified.

**Fig. 1:**
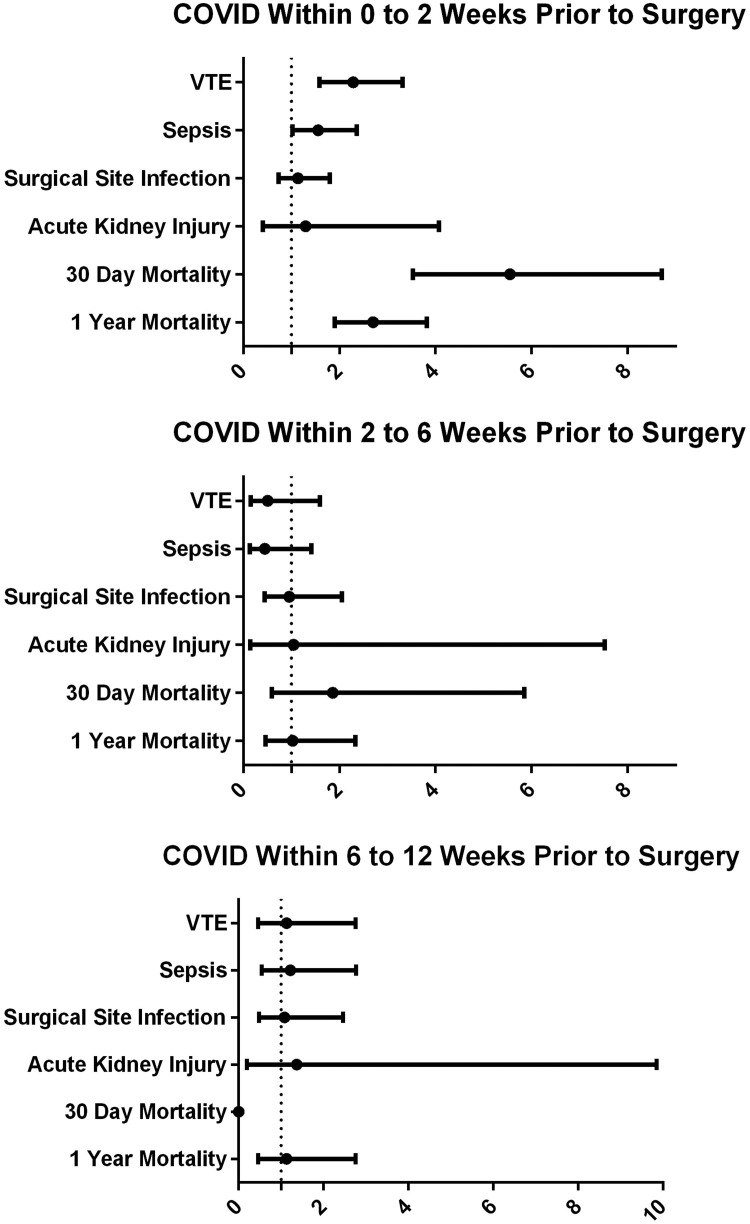
Odds for postoperative complications in patients with a confirmed COVID-19 diagnosis compared with controls without a positive COVID-19 test for the 90 days before surgery. Error bars represent 95% confidence interval.

Odds ratios for each perioperative complication as compared with patients who did not have a positive COVID test during the 90 days before surgery are plotted in Fig. 1. Risk for venous thromboembolic events was increased for patients who underwent surgery within 2 weeks of their COVID-19 diagnosis (OR 2.29, 95% CI 1.58–3.32) but not those between 2 and 6 weeks (OR 0.87, 95% CI 0.36–2.12) or between 6 and 12 weeks (OR 0.68, 95% CI 0.22–2.14) of COVID-19 diagnosis. The risk for sepsis was increased for COVID-19 diagnosis within 2 weeks of surgery (OR 1.56, 95% CI 1.03 to 2.36), but not between 2 and 6 weeks (OR 0.92, 95% CI 0.41–2.07) or 6 and 12 weeks (OR 0.59, 95% CI 0.19–1.86). The risk of 30-day mortality was increased for COVID-19 diagnosis within 2 weeks of surgery (OR 5.55, 95% CI 3.53–8.71), but not between 2 and 6 weeks (OR 1.86, 95% CI 0.59–5.85) or 6 and 12 weeks (no occurrences). The risk of 1-year mortality was increased for COVID-19 diagnosis within 2 weeks of surgery (OR 2.70, 95% CI 1.91–3.82), but not between 2 and 6 weeks (OR 1.03, 95% CI 0.46–2.34) or 6 and 12 weeks (OR 1.13, 95% CI 0.46–2.76) (Tables 2 and 3).

**Table 2:** Comparison of perioperative complication rates between COVID-19 positive and COVID-19 negative cohorts

Complication	COVID-	COVID+ 0-2		COVID+ 2-6		COVID+ 6-12	
	n *(%)*	n *(%)*	p*-value*	n *(%)*	p*-value*	n *(%)*	p*-value*
VTE	1,322 (3.52)	31 (8.07)	<.001	≤20^a^	.33	≤20^a^	.983
Sepsis	1,474 (3.94)	24 (6.14)	.048	≤20^a^	.233	≤20^a^	.243
Surgical site infection	1,648 (4.43)	≤20^a^	.645	≤20^a^	.9235	≤20^a^	.9811
Acute kidney injury	217 (0.56)	≤20^a^	.9071	≤20^a^	.64	≤20^a^	.7852
30-d mortality	370 (0.96)	21 (5.33)	<.001	≤20^a^	.4947	≤20^a^	.5026
1-y mortality	1,321 (3.52)	36 (9.5)	<.001	≤20^a^	.8953	≤20^a^	.9819

Values are reported as n (%). COVID-: COVID-19 negative, COVID+ 0-2: COVID-19 diagnosis within 0 to 2 weeks of surgery, COVID+ 2-6: COVID-19 diagnosis within 2 to 6 weeks of surgery, COVID+ 6-12: COVID-19 diagnosis within 6 to 12 weeks of surgery.

^a^ In accordance with N3C publication guidelines, individual values less than or equal to 20 are reported as ≤20 to protect subject anonymity.

**Table 3:** Association between COVID-19 and perioperative complication risk

Complication	COVID+ 0-2		COVID+ 2-6		COVID+ 6-12	
	*Odds ratio*	p*-value*	*Odds Ratio*	p*-value*	*Odds Ratio*	p*-value*
VTE	2.29 (1.58– 3.32)	<.001	0.51 (0.16–1.59)	1.316	1.13 (0.46–2.76)	.801
Sepsis	1.56 (1.03– 2.36)	.035	0.45 (0.14– 1.42)	1.233	1.22 (0.54– 2.77)	.646
Surgical site infection	1.14 (0.73–1.8)	.581	0.96 (0.45–2.06)	1.073	1.08 (0.48–2.46)	.864
Acute kidney injury	1.3 (0.41–4.07)	.667	1.05 (0.15–7.52)	.965	1.37 (0.19–9.85)	.767
30-day mortality	5.55 (3.53–8.71)	<.001	1.86 (0.59–5.85)	.293	n/a	n/a
1-y mortality	2.7 (1.91–3.82)	<.001	1.03 (0.46–2.34)	.948	1.13 (0.46–2.76)	.801

Values are reported as odds ratio (95% confidence interval). COVID-: COVID-19 negative, COVID+ 0-2: COVID-19 diagnosis within 0 to 2 weeks of surgery, COVID+ 2-6: COVID-19 diagnosis within 2 to 6 weeks of surgery, COVID+ 6-12: COVID-19 diagnosis within 6 to 12 weeks of surgery.

There was no significant difference in the rates of acute kidney injury within 2 weeks of surgery, (OR 1.30, 95% CI 0.41–4.07), between 2 and 6 weeks (OR 1.05, 95% CI 0.15–7.52), or between 6 and 12 weeks (OR 1.37, 95% 0.19–9.85). There was also no significant difference in the rates of surgical site infection within 2 weeks of surgery (OR 1.14, 95% 0.73–1.80), between 2 and 6 weeks (OR 0.96, 95% CI 0.45–2.06), or between 6 and 12 weeks (OR 1.08, 95% CI 0.48–2.46).

## Discussion

Using N3C, we assembled a large nationally representative cohort of patients undergoing lumbar spine fusion after a documented COVID-19 infection. Our findings indicate that patients undergoing surgery within 2 weeks from the initial COVID-19 diagnosis are at significantly increased risk for perioperative venous thromboembolic events, sepsis, and mortality, and this risk may warrant the postponement of nonurgent, elective spine surgery.

These results are consistent with the growing body of literature regarding the pathophysiology of COVID-19. There is evidence that COVID-19 creates a hypercoagulable state, which may be due to direct invasion of endothelial cells, as well as thromboinflammation and platelet activation [10], [11], [12]. Rates of venous thromboembolic events were significantly increased in patients hospitalized with COVID-19 infection, particularly in those requiring admission to an intensive care unit [13,14]. Studies employing routine duplex ultrasound of bilateral lower extremities in patients with severe COVID-19 found rates of DVT as high as 69%, although these studies were done in critically ill patients, which would be unlikely to be undergoing lumbar spine surgery except in cases of trauma [15,16]. Among nonhospitalized patients with COVID-19, no significant difference in symptomatic venous thromboembolic events has been observed [17,18]. The combination of spine surgery, which creates a hypercoagulable state by itself, combined with decreased mobility postoperatively and a COVID-19 infection likely all contribute to the elevated risk of venous thromboembolic events seen in our study.

The N3C cohort has previously been used to study patients undergoing orthopedic procedures. A study by Levitt et al. [19] of patients who underwent surgical treatment of hip fractures found an elevated 30-day mortality rate of 14.6% in patients who underwent surgery within the 7 days before or up to 30 days after a COVID diagnosis, versus 3.8% in the COVID-19 negative group.[] This is congruent with the increased 30-day mortality seen in our lumbar spine fusion patients who underwent surgery within 0 to 2 weeks after COVID diagnosis. Notably, the Levitt et al. [19] study cohort included patients from March through December of 2020. Our study builds upon this with over 2 years of data, from September 2020 through March 2023, and includes patients who received COVID-19 vaccinations as well. A second study using the N3C cohort, by Pitts et al. [20], studied patients undergoing ankle fracture fixation and found that patients who underwent surgery within the 7 days before or 30 days after a COVID-19 inpatient hospitalization had increased 30-day mortality rates but found no difference in rates of surgical site infection, acute kidney injury, deep vein thrombosis, or sepsis [20]. The lack of difference in nonmortality complication rates may be attributed to the fact that ankle fracture fixation is an inherently less invasive surgery than lumbar fusion and is typically an outpatient procedure. It is also important, however, that the Pitts et al. [20] study included patients undergoing surgery up to 30 days after a COVID-19 diagnosis. Our study suggests that COVID-19 is associated with increased risk for venous thromboembolic events and sepsis only when the surgery occurs within 2 weeks after COVID-19 diagnosis, and that without the granularity in time points, this effect may not be apparent.

The time-dependent association between COVID-19 infection and postsurgical outcomes has been previously explored by Bryant et al. [21] in a single-institution retrospective study of surgeries from all specialties which showed an approximately 1% reduction in risk for postoperative cardiovascular complications including deep venous thrombosis, pulmonary embolism, stroke, and myocardial infarction for every additional 10 days that surgery was delayed after a COVID-19 diagnosis.[] In contrast to our study, which did not show persistent increases in complication rates beyond 2 weeks after COVID-19 diagnosis, the temporal effect noted in the Bryant et al. [21] study persisted for the entire study observation period up to 600 days after COVID-19 diagnosis and was therefore unable to recommend an optimal timing of surgery after a COVID-19 infection. Another study, by Forlenza et al. [22], investigated postoperative outcomes of hip and knee arthroplasty after COVID-19 infection and also found a time-dependent effect with greater incidence of complications the closer in time the COVID-19 diagnosis was to the surgical procedure.[] Notably, this study found that the odds ratio for deep venous thrombosis and pulmonary embolism decreased with greater time following COVID-19 infection, but still remained elevated for up to 3 months. One potential explanation for this difference compared with our study's findings is again the study dates. The Forlenza et al. [22] study included patients undergoing surgery between January 2018 and April 2020, before the widespread availability and adoption of COVID-19 vaccination, which could potentially limit the duration that COVID-19 affects postoperative outcomes.

It is also worth noting that the effects of COVID-19 are variable in different patient populations. While some young and healthy patients suffer only mild symptoms and make a full recovery within weeks, other elderly patients with comorbidities may have prolonged declines in cardiopulmonary function, kidney failure, elevated stroke risk, fatigue, or cognitive difficulty [23,24]. Although we did not observe a significant age difference between groups in our cohort, especially between patients with and without COVID-19, it is limited in that we did not directly explore the effect of age on risk profile. We also lacked the data to stratify patients based on the number or type of COVID-19 vaccinations received, and as new vaccinations are constantly being developed to combat newly arisen strains, they may very well have a significant effect on postoperative complications after surgery by modulating the body's immune responses. Our study population also had significant differences in the proportion of patients with diabetes and hypertension between the COVID-19 infection group and the control group. Although these comorbidities may have a confounding effect on postoperative complications, we did not see any significant difference in complication rates in patients who underwent surgery between 2 and 6 or 6 and 12 weeks after COVID-19 infection. This suggests that the difference in complication rates seen was not due to a time-constant variable including chronic medical comorbidities. Future studies would benefit from randomization to eliminate these variables more definitively. Our study is additionally limited due to being retrospective in nature, and prospective studies would certainly be warranted in order to create strong evidence-based guidelines.

None of the increase in complication risks that we found, however, persist beyond 2 weeks, suggesting that 2 weeks may be the minimum amount of time necessary to postpone surgery after a COVID-19 infection is discovered. For more urgent cases where surgery cannot be safely postponed and must be undertaken within the 2 weeks following a COVID-19 diagnosis, a more aggressive VTE chemoprophylaxis regimen may be considered, although further study is necessary to fully answer the question of anticoagulation following spine surgery.

## Declarations of competing interest

One or more of the authors declare financial or professional relationships on ICMJE-NASSJ disclosure forms.

## Appendix Supplementary materials

Image, application 1

Image, application 2
