# Supplementary material for: A temporal analysis of perioperative complications following COVID-19 infection in patients undergoing lumbar spinal fusion: When is it safe to proceed?
Source: N Am Spine Soc J. 2023 Aug 11;16:100262. doi: 10.1016/j.xnsj.2023.100262 (PMC10504527; doi:10.1016/j.xnsj.2023.100262)
Supplement: Supplementary file 1 [file mmc1.docx]

| Lumbar Spinal Fusion | 22533, 22534, 22558, 22585, 22612, 22614, 22630, 22632, 22840, 22851, 22849 |
| --- | --- |

**Supplementary Table 1:** CPT Codes for lumbar spinal fusion procedure:
