# Supplementary material for: A temporal analysis of perioperative complications following COVID-19 infection in patients undergoing lumbar spinal fusion: When is it safe to proceed?
Source: N Am Spine Soc J. 2023 Aug 11;16:100262. doi: 10.1016/j.xnsj.2023.100262 (PMC10504527; doi:10.1016/j.xnsj.2023.100262)
Supplement: Supplementary file 2 [file mmc2.docx]

| Venous Thromboembolic Events | I82.4, I82.40, I82.401, I82.402, I82.403, I82.409, I82.49, I82.491, I82.492, I82.493, I82.499, I82.4Y, I82.4Y1, I82.4Y2, I82.4Y3, I82.4Y9, I82.4Z, I82.4Z1, I82.4Z2, I82.4Z3, I82.4Z9, I82.62, I82.621, I82.622, I82.623, I82.629, I26, I26.0, I26.01, I26.09, I26.90, I26.93, I26.99, I74, I74.0, I74.01, I74.09, I74.1, I74.10, I74.11, I74.19, I74.2, I74.3, I74.4, I74.5, I74.8, I74.9 |
| --- | --- |
| Sepsis | B37.7, B37.7, B37.700, B37.7, A41.802, A41.101, A41.504, A41.807, A26.7, A26.7, A26.7, A26.700, A54.86, A41.50, A41.805, A32.7, A32.7, A32.7, A32.700, A41.59, A41, A41, A41, A41.800, A41.8, A41.8, A41.8, A41.89, A40.8, A40.800, A40.8, A40.8, O98.801, A41.505, O85, 670.2, O85.x00, O85, O85, 670.22, 670.24, 670.2, A02.100, A02.1, A02.1, A02.1, 995.91, T81.411, A41.4, A41.400, A41.4, A41.4, A41.81, A41.51, A41.3, A41.300, A41.3, A41.3, A41.02, A41.01, A41.5, A41.500, A41.5, A41.5, A41.100, A41.1, A41.1, A41.1, A41.52, A41.53, A41.000, A41.0, A41.0, A41.0, A40.0, A40.0, A40.000, A40.0, A40.1, A40.1, A40.100, A40.1, A40.200, A40.2, A40.2, A40.3, A40.3, A40.300, A40.3, A41.2, A41.2, A41.200, A41.2, O86.04, T81.44, T81.44XA, T81.44XS, T81.44XD, O03.87, O08.82, O07.37, O03.37, O04.87, A41.901, P36.500, P36.5, P36.5, P36.5, P36.4, P36.4, P36.4, P36.400, P36.3, P36.300, P36.3, P36.3, P36.1, P36.1, P36.100, P36.1, P36.39, P36.19, P36.200, P36.2, P36.2, P36.2, P36.0, P36.0, P36.000, P36.0, P36.30, P36.10, A41.9, A41.9, A41.900, A41.9, 771.81, 995.92, R65.2, R65.20, R65.21, P36.301, P36.101, A40, A40, A40, A40.9, A40.9, A40.900, A40.9, P36.000, P36.0, P36.30, P36.10, A41.9, A41.9, A41.900, A41.9, 771.81, 995.92, R65.2, R65.20, R65.21, P36.301, P36.101, A40, A40, A40, A40.9, A40.9, A40.900, A40.9 |
| Infection | T81.42, T81.42XA, T81.42XS, T81.42XD, T81.41, T81.41XA, T81.41XS, T81.41XD, D998.3, D998.31, D998.32, D998.33, T81.3XA, T81.3XD, T81.3XS, T81.31XA, T81.31XD, T81.31XS, T81.32XA, T81.32XD, T81.32XS, T81.33XA, T81.33XD, T81.33XS |
| Kidney Injury | N17, N14, R39.2, D59.3, K76.7, T79.5, R39.2, 90.4, N99.0, N17.0, N17.1, N17.2, N17.8, N17.9, N19, 584, 584.5, 584.6, 584.7, 584.8, 584.9 |

**Supplementary Table 2:** ICD Codes for Complications
